# The Evolving Role of Radiation Oncology in the Management of Uterine Cervical Carcinoma: A State-of-the-Art Review for Non-Radiation Oncologists

**DOI:** 10.3390/life15121883

**Published:** 2025-12-10

**Authors:** Christian Haydeé Flores-Balcázar, Shuhey Augusto Matsumoto-Palomares, Diego Iván Chávez-Zaldívar, Adamary Itai Marin-Trinidad, Francisco Gerardo Castro-Pérez, Lucely del Carmen Cetina-Pérez

**Affiliations:** 1 Radiotherapy and Medical Physics Service, Instituto Nacional de Ciencias Médicas y Nutrición Salvador Zubirán, Mexico City 14080, Mexico; christian.floresb@incmnsz.mx (C.H.F.-B.);; 2 Radiotherapy Department, Instituto Nacional de Cancerología, Mexico City 14080, Mexico; 3 Clinical Research Department, Instituto Nacional de Cancerología, Mexico City 14080, Mexico

**Keywords:** cervical cancer, adjuvant radiotherapy, definitive radiotherapy, brachytherapy, chemoradiotherapy

## Abstract

Cervical cancer is one of the most common gynecological tumors globally. When diagnosed, treatment decisions should be based on a risk–benefit analysis of each treatment modality to obtain a cure with minimum complications. The optimal approach for management should consider clinical factors such as age, menopausal status, medical comorbidities, histological type, tumor size, and the extent of disease. Radiotherapy is the cornerstone for successful management in almost all clinical stages of this disease. Options for primary treatment in patients with early cervical cancer may include radical hysterectomy, fertility-sparing surgery, and postoperative radiotherapy with or without platinum-based chemotherapy (CT) according to pathology specimen findings. For locally advanced cervical cancer, chemoradiotherapy has been the standard of care based on the results of clinical trials that showed an overall survival (OS) advantage when adding cisplatin to radiotherapy. After chemoradiotherapy, a cervical boost is mandatory for increased local control and better survival. For metastatic or recurrent cervical cancer, the treatment approach is tailored according to symptoms and performance status. As many techniques and new technologies are available to decrease toxicity while improving the therapeutic ratio, it becomes necessary to collate the current evidence that most effectively enables clinicians to make informed decisions in the management of cervical cancer patients.

## 1. Introduction

**Figure 1:**
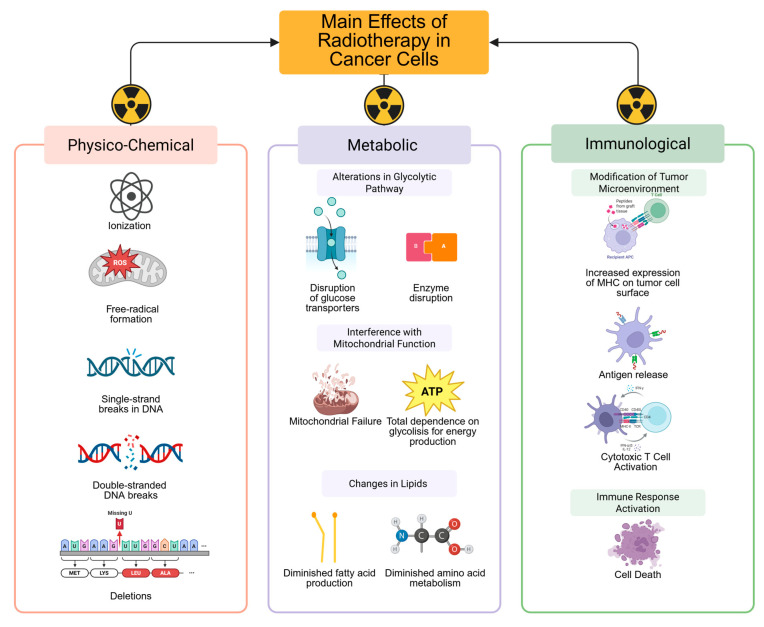
The administration of ionizing radiation to tissue causes direct damage to DNA by producing single-strand breaks, deletions, double-strand breaks, and indirect DNA damage due to the production of free radicals. This action leads to disruption of glucose transporters and enzymes involved in the glycolytic pathway, in addition to interference with mitochondrial function, thereby reinforcing cancer’s dependence on glycolysis for energy production. Moreover, radiation affects the production of essential fatty acids necessary for storing energy and amino acid metabolism, which are indispensable for cancer cells’ response to radiation. The metabolic effects of radiation ultimately modify the tumor microenvironment by increasing the expression of major histocompatibility complex (MHC) molecules on the tumor cell surface and releasing antigens, enabling cytotoxic T cells to easily recognize the tumor and activate an effective immune response, with subsequent cell death. The immune effects of radiotherapy synergize with additional therapies, such as CT and immunotherapy, to enhance the effectiveness of CC treatment. Created in BioRender. Flores, C. (2025) https://app.biorender.com/illustrations/67f6b86f3df04edb3a090179?slideId=a225dd13-e350-49da-80e7-3985b3c59de9.

Cervical cancer (CC) is the fifth most commonly diagnosed tumor after breast, prostate, colorectal, and thyroid cancer in Mexico, with 10,348 new cases reported in 2022 and a 5-year prevalence of 33,441 cases [1]. Globally, most new cases and deaths occur in low- and middle-income countries, making this neoplasm a public health threat that warrants a global strategy to achieve eradication. Despite the fact that the WHO has established a plan for the eradication of this neoplasm through screening campaigns and Human Papillomavirus (HPV) vaccination, CC cases continue to rise worldwide [2]. Against this backdrop, a structured approach is necessary for first-contact physicians to recognize the disease and promptly refer patients to an experienced cancer treatment center for accurate diagnosis and management. This approach should also aid in informing physicians about available treatments for treating CC, such as radiation therapy. Among general practitioners, knowledge of cancer-related symptoms has been reported in roughly 55% of those surveyed. However, the role of radiation therapy in cancer management, either for curative or palliative purposes, in addition to the management of radiation-induced adverse effects, is poor, primarily due to communication gaps and professional development needs. These gaps have a significant impact on patient care and outcomes [3]. Since the early days of the radiation oncology discipline, the utility of radiotherapy has been demonstrated in all CC disease spectrums, from early to recurrent and metastatic disease [4]. Radiation therapy is a treatment modality used for managing cancer that destroys tumor cells via ionizing radiation. It is primarily delivered with photons produced in a linear accelerator, although particles, such as protons, can also be used to treat gynecological cancers (Figure 1) [5,6,7].

The integration of radiation therapy for the management of any stage of CC is usually decided in a tumor board meeting, a collaborative setting in which all of the disciplines involved in the management of this neoplasm, from psycho-oncologists, nurses, and social workers to medical oncologists, gynecology oncologists, surgical oncologists, palliative care physicians, and radiation oncologists, work together to determine the most appropriate treatment plan and sequencing for each patient [8]. Multidisciplinary tumor boards are a minimum standard requirement outlined in international guidelines for best patient management. Adherence to tumor board meeting decisions directly relates to compliance with care standards, thereby improving patient management overall [9]. In this article, we aim to review current indications for radiotherapy, treatment modalities, and emerging topics regarding radiotherapy for CC. Since radiotherapy is the cornerstone for the successful management of this disease, it is necessary to summarize growing evidence and describe available techniques. This detailed information will aid non-radiation oncologists in making effective decisions in the management of cervical cancer patients.

## 2. Integration of Radiation Therapy in Early-Stage CC Management

Based on FIGO 2018 staging, early-stage CC is a tumor confined to the cervix of <4 cm in its greatest extent (Stages IA1 with lymph–vascular space invasion [LVSI], IA2, IB1, IB2, and IIA1). The main objective of treatment in the early stages of disease is to avoid locoregional spread (Table 1). Although disease management is surgical in nature, there are scenarios in which radiotherapy is recommended: (1) As adjunctive to optimal surgical management if risk factors for recurrence are found in the pathology specimen, (2) after a suboptimal surgical procedure when additional surgery cannot be performed, or (3) as a definitive single modality treatment for patients considered inoperable because of medical comorbidities [10].

### 2.1. Postoperative Radiotherapy After Optimal Surgical Management

Four to six weeks after surgery, pelvic radiotherapy should be considered when the final pathology report suggests a risk of locoregional recurrence. Based on the results of randomized controlled trials, prognostic histological features are classified as high- or intermediate-risk factors for recurrence. Peters et al. defined positive pelvic lymph nodes, positive margins, or microscopic parametrium involvement after radical hysterectomy and pelvic lymph node dissection as high-risk factors for recurrence in the GOG 109/SWOG 8797/RTOG 9112 trial [11]. In this phase III study, researchers assessed the role of adjuvant chemoradiation versus radiation alone in this group. The experimental arm included four cycles of CT with cisplatin and 5-Fluorouracil with pelvic conformal radiotherapy with a dose of 43.9 Gy in 29 fractions. The standard arm comprised only pelvic conformal radiotherapy to a dose of 43.9 Gy in 29 fractions. Vaginal vault brachytherapy (BT) was not employed. The 4-year OS rate increased from 71% to 81% with the addition of CT, and the 4-year progression-free survival (PFS) rate increased from 63% to 80% [11]. A trial assessing the sequence of CT was published in 2021. In the STARS trial, patients characterized by high-risk factors were randomized to adjuvant radiotherapy only, sequential chemoradiotherapy with cisplatin and paclitaxel, or concurrent chemoradiotherapy with cisplatin. With a 3-year disease-free survival endpoint, the sequential chemoradiotherapy group with cisplatin and paclitaxel was superior to concurrent chemoradiotherapy with cisplatin, providing a 5% benefit. However, this trial was limited to the Chinese population; thus, more information is needed to translate these results to Western populations [12]. With respect to vaginal vault BT, retrospective findings demonstrated benefit in decreasing local recurrence with no added benefit in survival for individuals receiving BT [13].

Intermediate-risk factors for recurrence after radical hysterectomy and pelvic lymph node dissection were defined by the GOG-49 and GOG-92 randomized trials. These features include lymphovascular space invasion (LVSI), maximum tumor diameter > 4 cm, and deep cervical stromal invasion (>1/3). The presence of one or more intermediate-risk factors confers a ≥30% three-year recurrence rate, and adjuvant pelvic external beam radiotherapy (EBRT) alone could be employed as an additional treatment modality [14,15]. The GOG-263 trial is the most recent publication on this topic; in this trial, patients with intermediate-risk factors were randomized to either conformal (cEBRT) or intensity-modulated radiotherapy (IMRT) with 50.4 Gy in 28 fractions alone or concurrent chemoradiotherapy with cisplatin and cEBRT or IMRT with 50.4 Gy in 28 fractions. Vaginal vault BT was prohibited in this trial. The findings demonstrated that treatment with cisplatin did not improve the 3-year recurrence-free survival rate over radiotherapy alone (88.5% vs. 85.4%, hazard ratio 0.698, 95% CI 0.408–1.192, *p* = 0.09), with OS rates also showing no favorable improvements. However, the findings do suggest that using pelvic IMRT without CT might be sufficient for local control [16]. To date, pelvic EBRT for patients with intermediate-risk factors remains controversial, as the results of a variety of prospective studies have failed to show a survival benefit with modest local control rates. The results of a recent meta-analysis demonstrated no benefit for local recurrence, risk reduction, or 5-year disease-free survival between adjuvant EBRT and observation groups [17]. The aim of the CERVANTES trial (NCT04989647) is to determine whether adjuvant pelvic radiotherapy, with or without CT and BT, confers a benefit in terms of lower recurrence or improved survival compared with no adjuvant treatment [18]. Vaginal cuff BT after adjuvant radiotherapy in patients with intermediate-risk factors after radical surgery has not translated into better survival benefits; thus, its use remains controversial [19].

### 2.2. Postoperative Radiotherapy After Suboptimal Surgical Management

While a simple hysterectomy is considered safe for well-documented FIGO 2018 stage 1A2 or 1B1 CC with a tumor ≤ 2 cm [10], cancer may be incidentally found in the pathology specimens of patients who have undergone a procedure for a benign disease; therefore, it is essential to conduct a thorough postoperative assessment. In a propensity score-matched analysis of the SEER database, the OS advantage of salvage radiotherapy over simple hysterectomy or radical surgery was assessed, with OS as the primary endpoint. The 5-year OS rate was higher in the salvage radiotherapy group (84.5%) than in the simple hysterectomy-only group (81.2%; HR 0.53, 95% CI 0.29–0.99, *p* = 0.046); in comparison, the 5-year OS was higher for patients with complementary radical surgery (77.9%) than for patients receiving salvage radiotherapy (73.2%). These results demonstrate that salvage radiotherapy is a viable option; however, when possible, complementary radical surgery should be performed to improve local control and survival [20]. When complementary radical surgery is not an option, chemoradiotherapy plus vaginal vault BT provides better 5-year PFS and OS than salvage radiotherapy alone [21]. It can therefore be concluded that postsurgical assessment of a patient with inadvertently diagnosed CC plays a significant role in determining the extent of the disease and influencing the choice of subsequent treatment, which may include completion of radical surgery and adjuvant therapy.

### 2.3. Definitive Radiotherapy

Both surgery and radiotherapy are valid options for managing early-stage CC, with equivalent results [22]. In cases in which surgery is not feasible, such as young but medically inoperable patients, elderly individuals with comorbidities, or those who refuse surgical treatment, external beam radiotherapy (EBRT) or concurrent chemoradiotherapy (CCRT) with BT boost can be considered. The choice of treatment is based on tumor characteristics, the severity of comorbidities, and menopausal status, all of which are key factors in determining the most suitable treatment. A study evaluating the prognostic implications of definitive radiotherapy was published in 2021. This study involved a propensity score analysis of the SEER database comparing hysterectomy with three cohorts of patients receiving radiotherapy for CC. Those in cohort A (1762 patients) were treated with EBRT +/− BT +/− CT, patients in cohort B (1244 patients) received EBRT and BT +/− CT, and those in cohort C (750 patients) received CCRT with BT. Among the three cohorts, cohort C produced in equivalent disease-free survival rates compared with hysterectomy, suggesting that patients who are not candidates for surgery should undergo CCRT plus a BT boost [23].

## 3. Integration of Radiation Therapy in Locally Advanced CC Management

Based on FIGO 2018 staging, a locally advanced tumor (LACC) refers to clinical stages IB3 and IIA2 to IVA, which encompass tumors > 4 cm in size and that extend beyond the cervix. The primary treatment objectives are to control locoregional disease and avoid systemic spread, which are crucial for achieving effective patient care. To achieve this objective, several forms of treatment have been evaluated over the years, from initial surgery to definitive CCRT to neoadjuvant chemotherapy and CCRT to the addition of different immunotherapy agents (Table 1). In a recent network Bayesian meta-analysis, researchers analyzed different forms of treatment for locally advanced cervical cancer. They analyzed PFS and OS following CCRT + BT, induction CT, neoadjuvant or adjuvant CT, EBRT alone, CCRT + BT with immune checkpoint inhibitors, or surgery [24]. For OS, CCRT + BT remained superior to EBRT + BT alone (HR 0.84; 95% CrI 0.67–1.06; superiority probability = 93.1%) and surgery (HR 0.75; 95% CrI 0.49–1.14; superiority probability = 91.4%). Concurrently, combinations involving immune checkpoint inhibitors or induction chemotherapy were associated with improved OS, though without statistically significant superiority over CCRT + BT. The PFS analysis results showed that CCRT + BT was superior to EBRT + BT alone (HR 0.76; 95% CrI 0.65–0.91) and induction chemotherapy followed by EBRT + BT (HR 0.69; 95% CrI 0.50–0.95). As above, no significant differences were found when comparing CCRT + BT with the addition of immune checkpoint inhibitors, induction CT followed by CCRT + BT, or adjuvant chemotherapy after CCRT + BT [24]. The results therefore demonstrate that CCRT with a BT boost is the most suitable treatment for locally advanced stages. This strategy offers an 8.4% advantage in locoregional control and a 7.5% survival advantage over EBRT + BT alone. CCRT + BT also improves complete responses by 10.2% compared with radiotherapy as the single treatment modality [25]. From the above evidence, it is clear that there is an urgent need to discuss topics related to radiotherapy treatment in patients with locally advanced CC, for which the literature provides scarce information.

### 3.1. Evidence Regarding External Beam Parametrial Dose Escalation

Historically, parametrial boost (EBPB) after CCRT has been used in patients with known lateral parametrial or pelvic side wall invasion to improve local control. The existing literature is largely retrospective, often involving older radiotherapy techniques, reporting improved local control when mean pelvic side wall doses of at least 56–60 Gy are delivered [26]. Boosting was performed with EBRT by means of midline block shielding; however, an increased incidence of complications involving the bladder, sigmoid, or rectum was reported [27,28]. Retrospective studies involving EBPB with image guidance showed that the parametrial boost correlated with reduced sexual and vaginal functioning [29]. The findings of recent studies evaluating EBPB demonstrated that omitting the parametrial boost did not compromise overall survival or local control [30]. From the above results, it is evident that EBPB should be discouraged, and a parametrial boost may be offered only when image-guided interstitial BT techniques are available.

### 3.2. Evidence Regarding Inguinal Node Irradiation in Patients with Lower One-Third Vaginal Invasion

The principle of elective bilateral groin irradiation in this scenario was extrapolated from vaginal and anal cancer management [31,32]. Retrospective data have shown that isolated inguinal recurrences in CC patients with inferior vaginal third invasion not receiving elective groin irradiation are rare, and, when compared with patients receiving inguinal radiotherapy, the latter modality causes unnecessary local skin damage and lower limb edema with no additional benefit to disease-free survival [33,34]. From this evidence, it is clear that in the absence of proven metastasis, groin elective radiotherapy is not recommended.

### 3.3. Evidence Regarding Elective Para-Aortic Nodal Irradiation

The para-aortic region is the most common site of nodal failure after pelvic CCRT. The results of a meta-analysis of six studies, of which five were retrospective, showed that pelvic wall involvement, bilateral pelvic node invasion, muscle involvement, >3 pelvic lymph nodes involved, pelvic node diameter ≥ 1.5 cm, bulky primary tumor, and high level of squamous cell carcinoma antigen are high-risk factors for para-aortic lymph node (PALN) metastasis. Iliac lymph node metastasis was the strongest predictor of PALN recurrence, suggesting that extended-field lymph node irradiation should be offered to such patients, since a 5-year OS rate and a 3-year DFS rate were noted [35]. Therefore, elective irradiation could be offered to patients with common iliac lymph node metastasis or >3 pelvic lymphadenopathy, preferably with intensity-modulated radiotherapy in a dose of 45 Gy in 25 fractions of 1.8 Gy. Further prospective trials on the effective dose of prophylactic elective para-aortic irradiation are required to maximize efficacy and minimize toxicity [36]. From these findings, elective para-aortic radiotherapy is recommended when PALN sampling has not been performed and one or more of the following conditions are present: Involvement of common iliac nodes, bilateral pelvic nodes, or three or more enlarged pelvic lymph nodes. If para-aortic sampling is conducted and the results are negative, elective irradiation is not necessary.

### 3.4. Evidence Regarding Radiotherapy Boost to Locoregional Metastatic Lymph Nodes

Patients with positive lymph node metastasis have shorter survival than those with early disease. The number and size of metastatic nodes are also predictors of poor prognosis. In a meta-analysis, researchers evaluated surgical debulking plus adjuvant radiotherapy vs. radiotherapy boost alone, showing equivalence regarding DFS. No difference was noted in lymph node, pelvic, and extra-pelvic recurrence between the treatment approaches. However, patients undergoing radiotherapy boost showed better OS than patients undergoing surgical debulking plus adjuvant radiotherapy [37]. It should be noted that most of the evidence presented in the literature is retrospective [38,39]. When advanced techniques are available, based on the results of the EMBRACE II study, metastatic locoregional lymph nodes with hypermetabolism on PET/CT scan, short axis > 10 mm on an image, or 5–10 mm with suspicious morphology should be boosted [40]. There is no consensus on the ideal dose for boosting lymph nodes; however, it is generally accepted as 55 Gy (EQD2) using intensity-modulated techniques and image guidance [41,42].

### 3.5. Evidence Regarding Neoadjuvant Chemotherapy to CCRT and BT in LACC

Due to the limited availability of radiotherapy equipment, many patients face lengthy waiting lists for the initiation of CCRT. This situation poses a significant concern due to the risk of disease progression. The INTERLACE trial was a prospective randomized pragmatic study in which the safety and efficacy of neoadjuvant chemotherapy were examined in patients with LACC. Following an LACC diagnosis, patients were randomized to six cycles of weekly carboplatin and paclitaxel before standard CCRT + BT or CCRT + BT alone. The findings of this study demonstrated a significant benefit in terms of PFS and OS with the implementation of neoadjuvant chemotherapy (NACT) before CCRT: the 5-year PFS rate was 73% with NC plus CCRT and 64% with CCRT alone (HR 0.65; *p* = 0.013); the 5-year OS rate was 80% and 72%, respectively (HR 0.61; *p* = 0.04) [43]. Despite its strengths, including being a pragmatic trial with substantial central revision of radiation planning quality and extended follow-up, the trial has some limitations, such as the use of outdated radiotherapy techniques. These techniques, combined with low marrow reserve related to neoadjuvant chemotherapy, prevented patients from receiving concurrent cisplatin due to high toxicity. In a landmark study published by Lindergaard et al., the authors compared the characteristics of patients and their treatment in the INTERLACE and EMBRACE-1 trials to form an “INTERLACE-like” cohort group comparable in T and N stage distributions [44]. For reference, the EMBRACE-1 trial was a prospective, observational, multicenter cohort study examining magnetic resonance imaging-based, image-guided adaptive brachytherapy in patients with locally advanced cervical cancer with excellent local control results [45]. When comparing the “INTERLACE-like” and EMBRACE-1 groups, the results obtained in the standard arm of INTERLACE were inferior to EMBRACE-I, thus suggesting that neoadjuvant chemotherapy could be compensating for suboptimal radiotherapy techniques when neither intensity modulated radiotherapy (IMRT) nor image-guided brachytherapy are used for the management of CC. In a recent meta-analysis on the use of NACT in LACC, treatment improved the objective response rate and the complete remission rate of patients but failed to improve overall survival and mitigate adverse effects [46]. Despite the strengths of the INTERLACE trial, including robust quality assurance of radiotherapy plans, a low dropout rate, and mature follow-up, several limitations hinder the application of its results to daily practice. These limitations include the use of conventional and non-standardized radiotherapy techniques, as well as the potential inability of patients to tolerate concurrent cisplatin-based chemoradiotherapy following neoadjuvant chemotherapy due to bone marrow toxicity. This raises important questions, such as whether certain patient groups do not benefit from neoadjuvant chemotherapy and whether the results of the INTERLACE trial can be applied to patients who are ineligible for cisplatin-based chemoradiotherapy. In situations where long waiting lists for the initiation of radiotherapy treatment are common, neoadjuvant chemotherapy can be considered for patients without para-aortic lymph node involvement to help prevent distant recurrences.

### 3.6. Evidence Regarding Adjuvant Chemotherapy to CCRT and BT in LACC

In the quest for the most effective treatment for women with LACC, researchers have examined the oncological outcomes of adjuvant chemotherapy (ACT) after CCRT. In the OUTBACK trial, a multicenter randomized controlled study, four cycles of carboplatin and paclitaxel after CCRT + BT were tested against the standard CCRT + BT. The primary endpoint of 5-year OS did not reach statistical significance, with rates of 72% in the adjuvant chemotherapy group and 71% in the CCRT + BT group (*p* = 0.81). Moreover, an elevated incidence of hematologic toxicity was noted in the adjuvant chemotherapy group [47]. In a recent network metanalysis comparing either NACT or ACT in patients with LACC, the authors did not find significant differences in PFS and OS [48]. From this evidence, at present, the practice of adjuvant ACT is not recommended.

### 3.7. Evidence Regarding Immunotherapy to CCRT and BT in LACC

The primary objectives of CCRT + BT in patients with LACC are to reduce tumor burden and achieve long-term disease remission while improving survival outcomes. However, most patients with LACC will suffer from local or distant recurrences. Based on the interaction of the human immune system with HPV, and to increase therapeutic efficacy, the addition of immune checkpoint inhibitors to CCRT + BT has shown strong results among patients with LACC. Immune checkpoint inhibitors act by restoring T-cell activity and regulating the PD-1/PD-L1 and CTLA-4 pathways, thereby enabling the immune system to activate an effective antitumor response [49]. Cisplatin, in addition to its direct cytotoxic action on tumor cells, also has a significant immunological effect, increasing the presentation of tumor antigens and the activation of cytotoxic CD8+ T cells [50]. The addition of radiotherapy to the management of CC involves several complex steps, from physical and chemical interactions to the production of inflammatory cytokines that facilitate tumor infiltration by T-cells, ultimately leading to immunogenic cell death [51]. Through its different modalities such as EBRT and BT, radiation achieves high tumor control by reducing doses to surrounding organs and subsequently diminishing acute and late toxicities. Among the various immunotherapy agents, Pembrolizumab, an anti-PD-1 blocker, has shown significant potential in the management of CC. In the ENGOT-cx11/GOG-3047/KEYNOTE-A18, a randomized, double-blinded, placebo-controlled Phase 3 trial published in 2024, researchers examined the effects of pembrolizumab addition to CCRT + BT against placebo and CCRT + BT alone, followed by pembrolizumab or placebo every 6 weeks for two years. At 24 months, the group treated with pembrolizumab demonstrated an improved PFS of 68% compared to the placebo group’s rate of 57% and improved OS, with rates of 83% vs. 75% for pembrolizumab and placebo, respectively [52]. However, the role of concurrent immunotherapy with Pembrolizumab is still early, and long-term data are not yet available. Based on the above evidence, it can be concluded that the use of pembrolizumab with CCRT should be considered for the management of patients with LACC and pelvic and para-aortic lymph node disease in settings where long waiting lists for radiotherapy initiation are not a problem and highly conformal techniques like IMRT are available.

## 4. Integration of Radiation Therapy in the Recurrent and Metastatic CC Scenarios

Disease management decisions are based on the functional status and the extent of disease spread, as OS and cervical cancer-specific survival differ among patients with oligometastatic or polymetastatic disease, in addition to those with lymph node metastases only versus those with visceral metastases [53]. For instance, patients with recurrent locoregional disease or distant oligometastases require intensive systemic and local management. In contrast, patients with widely disseminated disease may require symptom relief and supportive care offered by a specialized palliative care team. For both scenarios, radiotherapy has an established role.

### 4.1. Locoregional Salvage Reirradiation in Patients with Persistent or Recurrent Disease

Recurrence within the irradiated field occurs in up to 40% of patients with CC after CCRT [54]. The decision on whether to initiate reirradiation is multidisciplinary, and each patient should be evaluated individually, considering factors such as life expectancy, performance status, site for reirradiation, previous radiation dose, radiotherapy technique, and proximity to organs at risk [54]. For centrally located pelvic disease, exenteration is the procedure of choice. Reirradiation for peripheral recurrent or persistent disease can be performed using several techniques, such as high-dose interstitial BT, intracavitary BT, or SBRT, when patients are unsuitable for surgery or BT.

### 4.2. Radiotherapy to Metastatic Single-Site Distant Lymph Nodes in De Novo Stage IVB CC

Patients with single-site lymph node involvement have a better prognosis than those with multiple sites; the most common sites of distant metastasis are supraclavicular or inguinal [55]. As we continue to explore these sites, there has yet to be consensus on the RT dose and whether the target volume should include the entire nodal chain or only the involved node. However, the authors of retrospective series have reported acceptable local control rates with a dose ranging from 54 to 60 Gy to the distant site region, platinum-based chemotherapy, and pelvic control with radiotherapy [56].

### 4.3. Pelvic Radiotherapy in De Novo Stage IVB CC

Radiotherapy to the primary tumor after 4–6 cycles of doublet chemotherapy and after assessing the radiological response improves OS and PFS when compared to systemic therapy only. It reduces the morbidity associated with local progression (bleeding, pain, and invasion of adjacent organs). A dose of 45 Gy in 25 fractions of 1.8 Gy to the pelvis, with a boost to enlarged lymph nodes and an intracavitary BT tumor boost, offered a 3-year local control rate of 90% with a benefit in terms of overall survival, based on the results of a recent systematic review [57].

### 4.4. Oligometastases Management with Radiotherapy

The oligometastatic concept applies to patients with 1–5 metastases at diagnosis or recurrence, either in the visceral organs or in lymph nodes. Local control is offered with stereotactic body radiotherapy (SBRT) for such cases. Several retrospective series evaluating the role of SBRT in metastatic gynecological cancers have shown promising results, with 2-year local control rates ranging from 85% to 100%. These studies have correlated complete response to SBRT with increased overall and progression-free survival [58]. The largest retrospective cohort of SBRT in CC patients treated with 35 Gy in 5 fractions and a median BED10 of 59.5 Gy showed more favorable local control and progression-free survival rates in patients with a complete response after SBRT than in patients with any other response. Of note, the toxicity profile with SBRT was low [59,60]. Therefore, more research is required before it can be considered a routine option for recurrent/metastatic cervix cancer.

### 4.5. Radiotherapy and Immunotherapy Combination in Metastatic or Recurrent CC

Agents from the group of immune checkpoint inhibitors, including anti-PD-1, anti-PD-L1, and anti-CTLA-4 inhibitors, are among the most utilized drugs in metastatic CC. Their combination with radiotherapy modifies the tumor microenvironment, amplifies cellular inflammation, and activates tumor-specific effector T cells. However, the optimal sequence, dose, and fractionation are yet to be determined. Of note, SBRT is the most widely used radiation technique, along with immunotherapy, because of its ability to induce the regression of distant non-irradiated metastases through systemic immune activation, referred to as the abscopal effect [61].

### 4.6. Symptom Palliation in Advanced CC

The aim of prompt treatment is to provide early symptom control, reduce opioid requirements, and improve the quality of life of patients with vaginal discharge, bleeding, or pain. Short-course hypofractionated schemes, based on life expectancy, result in a substantial reduction in hospital visits. Commonly used schedules include 8–10 Gy in a single fraction, 20 Gy in five fractions, and 30 Gy in 10 fractions [62].

## 5. Choice of Radiation Therapy Technique for the Management of CC

Historically, the treatment of CC with radiotherapy was performed using bidimensional external radiotherapy, based solely on anatomical bony landmarks defined on X-ray plates using two opposite parallel fields. Bidimensional external radiotherapy was followed by using pendulum irradiation with cobalt-60 units to treat the pelvic lymph nodes in combination with radium-226 needles to the cervical tumor [63]. Subsequently, the ‘box’ technique, a significant advancement, was used to diminish the radiation dose to surrounding organs. It consisted of the administration of four irradiation fields with the anterior border placed anteriorly at the level of the pubic symphysis, the posterior border covering the sacrum at the level of S3–S4, and the superior limit at the level of the aortic bifurcation in the L3–L5 vertebral bodies or at the level of T12 in the case of an extended field when the para-aortic nodes were affected. The inferior border was defined as the lower limit of the obturator foramina; lastly, the lateral borders were placed 1.5–2 cm outside the pelvic ring. Although easily applicable, the use of standardized fields on bone structures is limited by the fact that they cannot be adapted to individual anatomic variations in patients, thus decreasing local control in areas outside the irradiation field [63]. Computed tomography (CT) has been used since the 1990s to plan and administer radiotherapy, marking the transition from two-dimensional to three-dimensional radiotherapy [64]. CT in radiation oncology provides anatomical and soft tissue information, in addition to volumetric dosimetry, which enables correlation with treatment results and toxicities. Using this information, the macroscopic gross tumor volume (GTV), clinical target volume (CTV), and planning treatment volume (PTV) were defined in reports 50 and 62 of the International Commission on Radiation Units (ICRU) [65]. Clinical stage and functional status play important roles in determining the appropriate radiotherapy technique and dose within CC management.

### 5.1. Conformal or Intensity-Modulated Techniques

Pelvic EBRT is currently delivered using different techniques: 3D conformal (cEBRT), intensity-modulated radiotherapy (IMRT), and volumetric arc techniques (VMAT). IMRT, with its superior organ protection compared to cEBRT in patients with gynecological malignancies, has been a game-changer. Since 2001, the results of numerous studies have demonstrated a decrease in acute gastrointestinal, genitourinary, and hematological toxicities using IMRT, without compromising tumor control. While highly conformal techniques are not mandatory, they are strongly recommended for treating CC [66].

### 5.2. External Beam Radiotherapy Dose for Definitive Treatment

Since the management of locally advanced CC relies on a combination of CCRT plus BT, multiple techniques have been used over the years to escalate dose, including the interposition of mid-pelvic blocks during EBRT delivery, fields of different sizes, or tumor boost prior to brachytherapy. However, whether a dose of 45 Gy or 50.4 Gy delivered in 1.8 Gy fractions should be administered remains unclear. Although retrospective, the authors of several studies have reported that 45 Gy in 25 fractions is the most advantageous scheme when combined with intracavitary brachytherapy [67]. This treatment regime not only enables dose-constraints to be reached more easily and reduces surrounding organ irradiation without compromising the total target area dose or clinical efficacy but also significantly decreases the risk of late gastrointestinal toxicity [68,69]. However, when IMRT is unavailable, resource-stratified guidelines suggest using cEBRT or a 4-field box technique [70].

### 5.3. Hypofractionated Pelvic Radiotherapy with Concurrent Chemotherapy

There are few publications on the use of hypofractionation in CC or other gynecologic malignancies. However, in some studies, researchers have reported promising results using 40 Gy in 15–16 fractions for pelvic radiotherapy, cisplatin chemotherapy, and intracavitary brachytherapy [71]. The HYPOCx-iRex Trial was a randomized phase 2 trial in which hypofractionated CCRT 44 Gy/20 fractions were compared with normofractionated CCRT with 45 Gy/25 fractions using IMRT and image-guided adaptive brachytherapy. Acute GI and GU toxicities were not significant, with similar local control or overall survival at the time of analysis [72]. These findings suggest that hypofractionated radiotherapy, combined with highly conformal techniques and image guidance, could offer significant benefits. While it is not yet recommended in CC patients outside a clinical trial environment, the potential advantages of this approach are certainly growing.

### 5.4. Point-A vs. Volume-Based Intracavitary Brachytherapy

The data on volume-based BT, obtained from prospective studies without comparator arms, is compelling. The results of published metanalyses, despite their heterogeneity, consistently demonstrate that volume-based BT reduces toxicity and improves survival rates compared to Point-A BT [73,74,75]. Such findings underscore the potential for volume-based BT to significantly enhance patient care, making it the preferred modality in CC patients.

### 5.5. Cervical Boost with Techniques Other than Brachytherapy After CCRT

Brachytherapy boost after CCRT significantly enhances clinical outcomes, particularly overall survival. However, the practicality of administering intracavitary brachytherapy can be hindered by patient-related factors such as unfavorable anatomy, contraindications for anesthesia, comorbidities, rejection of the procedure, or logistical challenges associated with the limited availability of equipment and financial constraints. Considering these challenges, alternative approaches have been actively explored. In a comprehensive review of CC treatment, focusing on patients who received a boost with brachytherapy, IMRT, or SBRT, the authors found no significant differences in overall survival between patients treated with SBRT and those treated with brachytherapy. The only exception was IMRT, which showed inferior overall survival when compared to brachytherapy [76]. Until randomized controlled trials with favorable results are published, it is advisable to refrain from using radiotherapy techniques other than BT for boosting the cervical tumor.

## 6. Conclusions

Radiotherapy, a crucial tool in the curative treatment of cervical cancer, can be employed in various ways. Whether it serves as definitive therapy or adjunctive to surgery, with or without chemotherapy, it should be integrated into a multidisciplinary approach. The information presented in this article is designed to enhance clinical practice and should be adapted to each radiotherapy center, respecting and considering their unique local resources.

## Figures and Tables

**Table 1:** Management according to clinical stage.

	Early Stage Stages IA1 with LVSI, IA2, IB1, IB2, and IIA1 (FIGO 2018)				Locally Advanced and Metastatic Disease Stages IB3 and IIA2 to IVB (FIGO 2018)			
	Adjuvant Radiotherapy		Definitive Radiotherapy		Adjuvant Radiotherapy		Definitive Radiotherapy	
	Medical Situation	Treatment Suggestion	Medical Situation	Treatment Suggestion	Medical Situation	Treatment Suggestion	Medical Situation	Treatment Suggestion
Radical Surgery	+ Lymph nodes + Surgical Margins Parametrial Invasion	CCRT 45 Gy with IMRT/VMAT and 6 applications of weekly cisplatin 40 mg/m^2^ and Image-Guided Intracavitary BT	Patient is a non-candidate or refuses surgery	IA1. Intracavitary BT IA2.-1B1 EBRT 45 Gy with IMRT/VMAT and Image-Guided Intracavitary BT. IB2-IIA1. CCRT 45 Gy with IMRT/VMAT and 6 applications of weekly cisplatin 40 mg/m^2^ and Image-Guided Intracavitary BT	Even when surgery is feasible in LACC with the addition of adjuvant CCRT, the most suitable management is definitive CCRT with a BT boost	CCRT 45 Gy with IMRT/VMAT and 6 applications of weekly cisplatin 40 mg/m^2^ + Immunotherapy and Image-Guided Intracavitary/Interstitial BT	IB3 IIA2 IIIA * IIIB	CCRT 45 Gy with IMRT/VMAT and 6 applications of weekly cisplatin 40 mg/m^2^ + Immunotherapy and Image-Guided Intracavitary/Interstitial BT
	LSIV, Tumor > 4 cm, Deep Stromal Invasion	EBRT 45 Gy with IMRT/VMAT and Image-Guided Intracavitary BT if + margins in vaginal cuff					IIC1 IIIC2 IVA ** IVB ***	CCRT 45 Gy with IMRT/VMAT with SIB 55Gy to lymph nodes > 1 cm and 6 applications of weekly cisplatin 40 mg/m^2^ + Immunotherapy and Image-Guided Intracavitary/Interstitial BT
	No risk factors for recurrence	No EBRT, periodic follow-up						
Suboptimal Surgery	Able to undergo complementary radical surgery	Management according to pathology results						
	Not suitable for complementary radical surgery	CCRT 45 Gy with IMRT/VMAT and 6 applications of weekly cisplatin 40 mg/m^2^ and Image-Guided Intracavitary BT						

* Consider including inguinal nodes in the radiotherapy field; ** confirm or rule out bladder/rectal invasion to aid in Brachytherapy decision-making; *** radiotherapy according to the burden of the disease and local symptoms. CCRT, concurrent chemoradiotherapy; IMRT, intensity-modulated radiotherapy; VMAT, volumetric arc therapy; BT, brachytherapy.

## Data Availability

No new data were created or analyzed in this study. Data sharing is not applicable to this article.
